# Long-term health effects of antipyretic drug use in the aging population: a systematic review

**DOI:** 10.25122/jml-2024-0081

**Published:** 2024-09

**Authors:** Seema Mahesh, Esther van der Werf, Mahesh Mallappa, George Vithoulkas, Nai Ming Lai

**Affiliations:** 1 Faculty of Health and Medical Sciences, Taylor’s University, Subang Jaya, Malaysia; 2 Centre For Classical Homeopathy, Bengaluru, India; 3 International Academy of Classical Homeopathy, Alonissos, Greece; 4 Homeopathy Research Institute, London, United Kingdom; 5 Bristol Medical School, University of Bristol, Bristol, United Kingdom; 6 University of the Aegean, Mytilene, Greece

**Keywords:** antipyretics, aged, systematic review, inflammation, fever, immunosenescence, acetaminophen, non-steroidal anti-inflammatory agents

## Abstract

It is unclear whether fever suppression in the elderly provides long-term benefits or poses risks due to their distinct immune profiles and body temperature regulation compared to younger individuals. This study aimed to assess the long-term health effects of antipyretic treatment during infections in the elderly. A systematic review was conducted, including studies that compared antipyretic treatment with other drugs, therapies, placebo, or no treatment. PubMed, Embase and Cochrane CENTRAL databases were searched. Primary and secondary outcomes were the onset or worsening of chronic inflammatory diseases, fever reduction, length of hospital stay, patient satisfaction, mortality, laboratory indicators of morbidity, and progression to complications, respectively. Out of 11,481 studies screened, 17 were included (two randomized controlled trials [RCTs], seven observational studies, one case series, and seven case reports). None investigated the primary outcome or patient-reported outcomes. The risk of bias in the included studies ranged from unclear to high. Due to the heterogeneity of the studies, a narrative synthesis was conducted, as meta-analysis was not feasible. Antipyretics showed a significant reduction of fever in RCTs. Five studies reported a significant drop in blood pressure, and one showed significant mortality from antipyretics. Morbidity indicators and length of stay were available only in the studies that reported adverse events. The certainty of evidence, assessed using GRADE, was low to very low for all outcomes. Evidence regarding the long-term benefit or harm from fever suppression with antipyretics during infections in the elderly is insufficient.

## INTRODUCTION

An undercurrent of chronic inflammation alters the immune responses as one ages [1,2], making infections more difficult to identify and more damaging than in younger individuals [3-7]. Elderly individuals (>60 years old) are known to have a lower baseline body temperature than the younger population, which is thought to be an adaptive mechanism for longevity [8-11]. It has been reported that the immune responses of older people are comparatively less effective than those of younger people, which may be detrimental to their outcomes in acute and severe illnesses [6,12,13]. On the other hand, there is evidence demonstrating the adverse effects of 'over-reaction' of the immune system and the protective effects of hypothermia in survival [14-16]. Until now, no conclusive answer exists on whether fever is beneficial or harmful [17-21]. However, fever is routinely suppressed to curb the extra metabolic and cardiorespiratory demand it imposes on the patient, as well as to reduce fever-associated discomfort [22-25]. Most of the time, this involves over-the-counter drugs without medical advice [23,26-28]. Some studies have linked the suppression of acute inflammation, including fever [29-34], to the onset and progression of chronic inflammatory diseases, potentially contributing to the global increase in such conditions [29,35]. However, there is no comprehensive review of the current evidence regarding the overall benefits and harm of treating fever in the elderly during infections. Based on the existing literature, we hypothesized that reducing fever during infections in the elderly may be detrimental in the long term, leading to the onset or worsening of chronic inflammatory diseases.

### Objectives

The primary objective was to synthesise available evidence to determine the long-term effects of fever suppression during infection in the elderly with antipyretic drugs. Our secondary objectives were to determine the effect of such treatment on fever reduction, length of hospital or intensive care unit (ICU) stay, patient satisfaction, mortality, changes in morbidity indicators, and progress to complications (including adverse events).

## Material and Methods

The study protocol was registered on PROSPERO (reg. no. CRD42020160854) and published on a Scopus-indexed publishing platform [36]. All additional data are available in an online data repository referenced in the data availability section.

### Literature search

**Figure 1:**
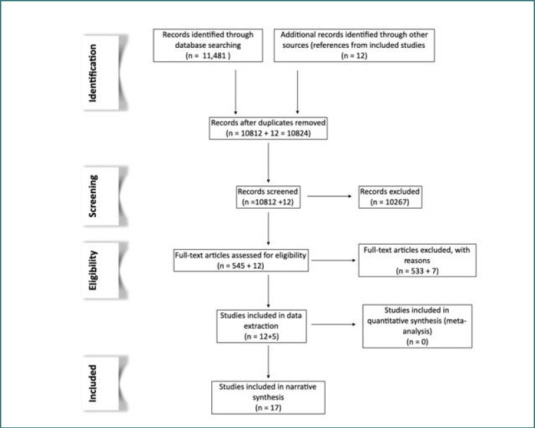
PRISMA chart depicting the inclusion-exclusion process of the review

A systematic search of PubMed, Embase, and Cochrane CENTRAL databases was conducted from their inception until March 27, 2021, using personalized search terms for each: medical subject headings (MeSH) terms for PubMed, Emtree terms for Embase and title/abstract terms for Cochrane CENTRAL (detailed search strategies are available in the online data repository). The screening and inclusion process is illustrated in a PRISMA flow chart (Figure 1). Two researchers independently reviewed and selected relevant studies based on the following criteria:

#### Inclusion criteria

- Studies involving elderly individuals receiving antipyretic medication for fever reduction during an infection.
- Comparisons with placebo, other drugs/therapies, or no treatment.
- Studies that included exclusively or predominantly the elderly population or studies that provided separate data for the elderly.
- No restrictions on study design or language.
- Human studies from peer-reviewed published literature.

#### Exclusion criteria

- Studies in which antipyretics were administered for reasons other than fever reduction, such as pain relief.

### Outcomes

#### Primary outcome

The onset or worsening of chronic inflammatory diseases following fever intervention, as defined by authors, with long-term effects defined as those persisting for more than three months after fever suppression.

#### Secondary outcomes

These included fever reduction, all-cause mortality, progression to complications, adverse events, length of hospital or ICU stay, changes in laboratory or radiological findings indicating morbidity, and patient satisfaction with the interventions.

### Data collection

Data from included studies were extracted using customized forms based on the Cochrane Effective Practice and Organization of Care (EPOC) guidelines [37], adjusted for the specific study types (online data repository). Information was gathered on publication details, study characteristics, sample sizes, control and intervention groups, blinding methods, types of infection, intervention specifics, inclusion/exclusion criteria, patient demographics, outcomes measured, follow-up periods, attrition rates, statistical populations (intention-to-treat or per protocol), and relevant statistical analyses. Any missing data were recorded as such, and the whole sample (intention to treat) was considered for analysis. A meta-analysis was planned if the data could be pooled, and in case the pooling was not possible, a narrative synthesis was planned.

Risk of bias (ROB) for randomized controlled trials (RCTs) was assessed using the Cochrane ROB tool, and for observational studies using the ROBINS-I tool [38,39]. For case series, the Joanna Briggs ROB tool was employed [40,41], and for case reports, the Cochrane Handbook’s recommended domains were followed [39] (online data repository).

The certainty of evidence was assessed using the Grading of Recommendations, Assessment, Development, and Evaluations (GRADE) guidelines, adapted for narrative synthesis [42-44]. The outcomes were assessed for risk of bias due to study design, inconsistency, imprecision, publication bias, residual confounding, large effect, and dose-response gradient.

### Data synthesis and analysis

Due to the heterogeneity of the effect measures across studies, the effect sizes could not be pooled for a meta-analysis. Where possible, data were transformed into means and standard deviations, and drugs were grouped if administered separately in different studies.

## RESULTS

Of the 11,481 studies identified through the initial search, 10,812 remained after deduplication. An additional 12 studies were identified through reference searches of the included studies. All references were managed using a reference management system [45]. Ultimately, 17 studies were included in the final analysis:

- 2 RCTs [46,47],
- 5 prospective observational studies [48-52],
- 2 retrospective observational studies [53,54],
- 1 case series [55], and
- 7 case reports [56-62].

Full-text screened studies, when excluded, were done so with explanations in the data extraction forms. Table 1 provides the characteristics of the included studies.

**Table 1:** Characteristics of studies included

Study ID	Country	Design	Setting	Population	Intervention	Comparator	Outcomes	Observation period	*n*	ROB
Cunietti 1993	Not mentioned	RCT	Not mentioned	≥65 Y viral/bacterial infections URTI/LRTI Fever >38°C informed consent	Nimesulide	Paracetamol	1. Fever reduction 2. Progress to complications, hemodynamic 3. Laboratory markers of morbidity	3 days	39	U
Reiner 1985	Not mentioned	RCT	Not mentioned	18 - 90 Y with fever	Nimesulide	Diclofenac, Placebo	Fever reduction	6 hours	81	U
Cantais 2016	France	Obs - P	ICU	Adult patients requiring IV acetaminophen infusion according to the attending physician’s judgment and having arterial pressure monitored via an arterial catheter	Paracetamol	Nil	1. Fever reduction 2. Progress to complications, hemodynamic 3. Mortality	3 hours	160	H
Hersch 2008	Israel	Obs – P	ICU	Critically ill patients in the ICU who were febrile (body temperature ≥ 38°C), ventilated, sedated, and experiencing sepsis	Propacetamol	Nil	1. Fever reduction 2. Progress to complications, hemodynamic	2 hours	14	H
Krajcova 2012	Czech Republic	Obs - P	ICU	>18Y, artificially ventilated and administered paracetamol, monitored by PiCCO, with sinus rhythm	Paracetamol	Ranitidine	1. Fever reduction 2. Progress to complications, hemodynamic	Until fever resolution occurred	6	H
Lee 2012	Korea and Japan	Obs - P	ICU	All adult patients requiring ICU >48 hours	NSAIDs, paracetamol	External cooling	1. Fever reduction 2. Mortality	28 days	1425	H
Poblete 1997	Switzerland	Obs - P	ICU	Patients undergoing mechanical ventilation in ICU with rectal temperature >38.5C and in whom the attending physician wished to reduce fever (Patients did not have inspired O2 fraction of >0.6; did not consume caloric intake exceeding energy expenditure calculated at baseline)	Propacetamol, metamizole	External cooling	1. Fever reduction 2. Progress to complications, hemodynamic 3. Mortality	Till metabolically stable state was achieved	20	H
Ye 2017	USA	Obs - R	ICU	All patients meeting criteria for sepsis, receiving mechanical ventilation, with fever and hypothermia	NSAIDs, paracetamol	External cooling	Mortality	Not specified	8711	H
Zhang 2015	USA	Obs - R	ICU	All patients meeting the criteria for sepsis	NSAIDs, paracetamol	External cooling	Mortality	Not specified	15268	H
Ban 2016	Korea	CS	Allergy clinic	Patients presenting with fever and blistering lesions with a history of acetaminophen exposure preceding the onset of symptoms	Paracetamol	Nil	1. AdEv 2. LOS 3. Laboratory marker of morbidity	Case 1: 67 days Case 2: 36 days	2	H
Akashi 1997	Japan	CR	Hospital	64- and 70-year-old with Acetaminophen-induced pneumonitis	Paracetamol	Nil	1. AdEv 2. Laboratory marker of morbidity	Case 1: 4 months Case 2: 3 days	2	H
Ayonrinde 2000	Australia	CR	Hospital	65-year-old with anaphylactoid reaction	Paracetamol	Nil	1. AdEv 2. Laboratory marker of morbidity	3 days	1	H
Danguy 2010	France	CR	Hospital	71-year-old with Acetaminophen-induced hypotension	Paracetamol	Nil	Progress to complications, hemodynamic	5 days	1	H
Gonzalo - Garijo 2006	Spain	CR	Hospital	68-year-old with Ibuprofen-induced fever	Ibuprofen	Nil	1. AdEv 2. LOS 3. Laboratory marker of morbidity	3 months	1	H
Kim 2014	Korea	CR	Hospital	60-year-old with TEN	Paracetamol	Nil	1. AdEv 2. LOS 3. Laboratory marker of morbidity	30 days	1	H
Kondo 1993	Japan	CR	Hospital	63-year-old with Acetaminophen-induced eosinophilia	Paracetamol	Nil	1. AdEv 2. Laboratory marker of morbidity	7 days	1	H
Prabhu 2005	Nepal	CR	Hospital	65-year-old with fixed drug eruption	Paracetamol	Nil	1. AdEv 2. Laboratory marker of morbidity	48 hours	1	H

RCT, randomized controlled trials; Obs P, prospective observational; Obs R, retrospective observational; CS, case series; CR, Case reports; U, unclear; H, high; ICU, intensive care unit; NSAIDs, non-steroidal anti-inflammatory drugs; LOS, length of stay; AdEv, adverse events; TEN, toxic epidermal necrolysis.

The review included 25,722 participants from 17 studies. The interventions assessed were acetaminophen, its derivatives, and NSAIDs, which were compared against each other, external cooling, placebo, or no treatment. Due to the following factors, a meta-analysis was not possible:

1. Heterogeneous study designs
2. Outcomes measured at different time points, using various techniques and reported in heterogeneous ways and
3. Non-uniformity in comparison between interventions.

A narrative synthesis was conducted as planned earlier, following the guidelines of the Cochrane Consumers and Communications Group [43] (online data repository) and the ESRC methods program [44]. The results were synthesized and reported in accordance with PRISMA and the SWiM protocol extension for narrative synthesis [63,64] (online data repository). The certainty of evidence was assessed using GRADE guidelines adapted for narrative synthesis [42]. A summary of findings is provided in the online data repository.

### Grouping by study types

The studies were grouped according to the study design to compare the effects. Below is a summary of all included studies according to the study type.

#### RCTs

Both included RCTs compared antipyretic drugs among themselves and with placebo for fever reduction and reported adverse events.

#### Observational studies

Four out of five prospective observational studies focused on cardiovascular effects and fever reduction with intravenous antipyretics. Two studies [50,52] investigated mortality from antipyretics. Lee *et al*. [52] further compared the effects of antipyretic therapy between patients with sepsis and non-sepsis, including fever temperature ranges and their association with mortality. Both retrospective studies investigated mortality from antipyretics and external cooling, comparing sepsis and non-sepsis patients. All observational studies involved ICU patients.

#### Case series and reports

The case series and case reports documented adverse events, including Stevens-Johnson syndrome (SJS), toxic epidermal necrolysis (TEN), pneumonitis, hypotension, eosinophilia, fixed drug eruptions, and aseptic meningitis.

### Risk of bias (ROB)

The two RCTs had an unclear ROB due to a lack of full-text availability, while the other studies showed high ROB due to study design, confounding, and missing data (Table 1).

### Narrative synthesis

None of the included studies investigated the primary outcome of onset or worsening of chronic inflammatory diseases due to antipyretic use. No studies examined patient satisfaction with antipyretic therapy either.

#### Fever reduction

Antipyretics effectively reduced fever in the RCTs, showing a mean reduction of 1.6 ± 0.42°C [46,47]. However, in critically ill patients and those with sepsis, fever reduction was less pronounced, ranging from 0.21 ± 1.15 to 0.69 ± 0.4°C in observational studies [49-52]. External cooling showed a similar effect, with fever reductions ranging from 0.2 ± 0.1 to 2 ± 1.44°C [49,52]. The case series did not contribute to this outcome, and only one case report mentioned a fever reduction after paracetamol administration (online data repository) [59].

#### Progress to hemodynamic complications

Seven of the 17 studies reported the development of hypotension, though the severity varied across studies (online data repository). One RCT documented a systolic blood pressure (SBP) drop of 3–4% [46], while prospective observational studies reported a mean arterial pressure (MAP) decrease ranging from 4.8 ± 12.28 to 12.59 ± 8.1 mmHg [48,50,51], except for one study that found no change in MAP [49]. Case reports also indicated varying degrees of MAP reduction [57,60].

Heart rate was also measured in four studies, and a trend towards reduction was observed after administering antipyretics, although this was not statistically significant [46,48,49,57]. One study found a non-statistically significant trend toward reduction in peripheral resistance and cardiac output from antipyretic drugs [48].

#### Mortality

No mortality cases were reported in studies involving common self-limiting viral or bacterial infections, as indicated by the RCTs and case reports (online data repository). However, in sepsis and ICU patients (reported in observational studies), mortality was positively associated with antipyretic therapy in the highest fever temperature range, with OR = 2.61 (95%CI, 1.11–6.11) and 2.05 (95%CI, 1.19–3.55) for NSAIDs and acetaminophen respectively (online data repository), when compared to external cooling (OR = 1.2; 95% CI, 0.70–2.05)] [54]. Mortality was not affected by antipyretic drugs in other temperature ranges and did not differ from the non-sepsis group [53,54].

When all the studies comparing overall mortality between antipyretic drugs and external cooling in sepsis patients were considered, the OR and 95%CI were 0.75 (0.64–0.87; *P* = 0.0003) [26,52,54]. However, mortality in the antipyretic drugs group compared to external cooling in the non-sepsis group was not different and yielded an OR of 0.30 (95%CI, 0.07–1.30; *P* = 0.11). Mortality in the antipyretic drugs group compared to non-treatment in patients with sepsis was higher but statistically non-significant (OR = 1.11; 95% CI, 0.96–1.28; *P* = 0.15).

#### Length of stay (ICU/hospital)

The case series reported an average ICU stay of 51.5 days due to adverse effects from antipyretic treatment [55]. Case reports showed a mean ICU stay of 4 days due to adverse effects from these drugs [56-62]. However, the observational study documenting the length of stay did not specifically assess the impact of antipyretic treatment on this outcome [52] (online data repository).

#### Morbidity indicators (laboratory and radiological findings)

Laboratory markers influenced by antipyretic drugs were reported only in case reports documenting adverse events [55-62] (online data repository).

#### Adverse events

Adverse events were reported in the RCTs, case series, and all case reports (online data repository). A total of 18 adverse events were documented across ten studies [46,47,55-62], including mild transitory effects, hypotension, Steven Johnson syndrome, toxic epidermal necrolysis, aseptic meningitis, pneumonia, and fixed drug eruption.

### Certainty of evidence

As assessed using the GRADE framework, the certainty of evidence was low for outcomes related to fever reduction and mortality and very low for all other outcomes (online data repository).

## Discussion

Recent research has challenged the traditionally accepted mechanism of antipyretic action, primarily thought to involve COX-2 inhibition, suggesting that these drugs may modulate the immune system more broadly [30,65]. The phenomenon of fever induction as a part of acute inflammatory response has been retained for over 600 million years of evolution despite its metabolic cost [66]. Upon tissue breach, the resident immune cells engulf the foreign material and release cytokines, which promote the production of prostaglandins. The prostaglandin PGE2 stimulates the preoptic area of the hypothalamus to upregulate the core body temperature, which we recognize as fever. This upregulation is a signal for the recruitment of immune cells like macrophages and neutrophils, which play essential roles in defending against pathogens. Innate and adaptive immunity components are programmed to respond to the febrile temperature to induce acute inflammation and neutralize the pathogens [67-71]. The pro-inflammatory cytokines at this stage are necessary to stimulate anti-inflammatory cytokines later. Each step of the acute inflammation initiation is necessary for the downstream resolution of the inflammation to occur efficiently. If the process gets hindered at any point, the tissue does not return to its normal state after the inflammation. For example, in the later stage of inflammation, PGE2, which is responsible for fever as already described, turns anti-inflammatory and promotes phagocytosis of the used-up neutrophils and their removal from the tissue environment. Failing this important function, the tissue remains mildly inflamed and never returns to normal. Antipyretics prevent the synthesis of the PGE2 by inhibiting the cyclo-oxygenase enzymes 1 and 2. Therefore, there is no signal to the hypothalamus to raise the alarm through fever, resulting in compromised defense and a compromised resolution of inflammation [32-34,72-77]. A low-grade constant inflammation results, which may eventually become chronic [29,78,79].

The activation of chronic inflammation from inhibition of fever has been recorded in some scenarios, but the evidence is unclear [80-85]. In our review, we aimed to provide clarity on this phenomenon. However, most included studies did not investigate long-term effects, as follow-up periods ranged from as short as two hours to a maximum of four months. Only two studies had follow-ups long enough to potentially assess long-term consequences, but neither made any observations or comments on this aspect. As a result, we found no conclusive evidence linking fever suppression with the onset or worsening of chronic inflammatory diseases, highlighting this as a significant knowledge gap, considering that many immunological studies have raised concern over this phenomenon [30,32,86].

While antipyretic use is common, in self limiting infections, they have been shown to reduce fever and alleviate symptoms [46,47,59], but the same benefits were not observed in patients with sepsis or critical illness [48-53,87]. In these severe cases, physical cooling appeared to provide little benefit, except in patients with severe sepsis who were sedated, where it was associated with decreased energy expenditure, fever reduction, and a lowered heart rate [49,52,54,87]. Additionally, there was a strong association between mortality and antipyretic therapy in sepsis patients experiencing high fever ranges [87]. The reason behind this was postulated by the investigators as resulting from the difference in the role fever plays in various subjects during different infection episodes with varying severity. In some cases, fever is protective, while in others, it may be harmful [52]. Available meta-analyses comparing active fever management to less aggressive intervention found no evidence to support routine fever suppression, even in physiologically compromised individuals [88]. Therefore, the best current recommendation is to assess each case individually, considering immune function and potential organ damage, before deciding whether to recommend or avoid fever suppression [89]. They also recommend closely monitoring for organ dysfunction and the presence of acute brain pathologies, coma, or cardiac arrest before determining the appropriate fever management regimen. The recommendation is permissive fever management in the absence of such pathologies or organ compromise, where the temperature is below 41°C. However, this recommendation is not based on high-quality evidence and is generalized to all cases of fever in the ICU, which needs further investigation.

Hypotension emerged as a notable adverse effect following the administration of antipyresis, as reported in seven studies [46, 48-51,60]. The study authors postulated that this may be due to a loss of peripheral resistance and decreased cardiac output [48]. Other adverse events, like pneumonitis, anaphylactoid reaction, SJS/TEN, fixed drug eruption, and aseptic meningitis, were rare but adequately reported in seven studies [51-59,61,62]. The length of stay and morbidity indicators were available but had little consequence.

Earlier systematic reviews examining the effect of antipyretic treatment on sepsis either found insufficient evidence to make a robust estimate regarding mortality or reported favorable outcomes for fever reduction [80,90-93]. However, they did not consider the elderly specifically or include non-randomized studies. This review focused on elderly individuals and included all study types to capture a broader range of evidence. While it is desirable to conduct a systematic review and meta-analysis of RCTs for their methodological superiority, a study aiming to assess the adverse effects of a drug may not find much evidence in them. The ethical/practical issues associated with conducting such studies make it necessary to include observational studies. The evidence is more likely to be better represented in anecdotal reports such as case series and case reports where the real-world situation is depicted better. The inclusion criteria for RCTs are usually very restricted, making the inclusion of case series, case reports, and observational studies imperative to obtain more generalizable evidence. However, the inherent limitations of these reports mandate a cautious approach in their assessment and incorporation into the body of evidence. In the end, with the scarce findings on the elderly population, the findings were similar to those in previous systematic reviews on the general population.

### Assessment of large studies

Three large studies included in this review [52–54] incidentally compared the effects of antipyretic drugs with external cooling. Lee *et al*. [52] examined both septic and non-septic patients, while Ye and Zhang focused exclusively on septic patients [53]. All three studies investigated mortality only. Forest plots of these studies (online data repository) suggest that there may be a slight increase in mortality in the antipyretic drug group compared to no treatment in septic patients, though this difference was not statistically significant. All three studies, however, show a significant increase in mortality from antipyretic therapy in general (combining external cooling and drugs) against non-treatment in septic patients. However, this effect seems to result mainly from external cooling than drugs. The certainty of evidence on mortality from these three studies is low as they are observational studies and have a glaring confounding factor that cannot be ruled out – it is possible that sicker patients were more likely to receive antipyretic therapy, automatically increasing the mortality. Thus, even considering the large studies included in this review, it is not possible to recommend or deter treatment of fever with antipyretic drugs in the elderly during infections. While individual studies found significant effects of antipyretic drugs on fever reduction, mortality, and the development of hypotension, when systematically synthesized, the certainty of the evidence was found to be low or very low.

### Strengths and limitations

The strength of this review lies in its inclusion of a wide range of study designs, including case reports and case series, which allows for a closer representation of real-world clinical situations and a broader coverage of different etiologies. However, the review faced some challenges, such as difficulty obtaining the full text of Reiner's study [47] and the original publication of Cunietti's work [46]. Poblete’s study also lacked explicit information separately for the elderly [49]. We had to work with the partial data that was available. Furthermore, the primary outcome of interest was not evaluated and reported in any study. We emphasize this as a research gap and advise a study assessing the long-term effects of antipyretic drugs in the elderly. As the review focuses on the harmful effects of an intervention, the use of narrative synthesis limits our ability to compare the effect quantitatively. This could not be avoided due to the heterogeneity of measures and study types. However, when conducted systematically as done here, adhering meticulously to the framework and followed by rigorous quality assessment of the evidence, narrative synthesis provides an overall picture of the outcomes, which is reliable to a great extent.

### Future research

With this review, the question of the long-term effect of antipyretic treatment in the elderly during infections was identified as a research gap, and considering the proclivity of such practice in the general population, we need to investigate this in the future. We identified issues in investigations that may be responsible for such confusion on the subject. The aging adult population has not been the focus of any quality study. This is essential as the immunological phenomena change with age, and what applies to the younger population may not apply to the elderly. Additionally, none of the studies included in this review had a long enough follow-up period to assess the long-term effects of acute fever or antipyretic treatment. This again, is an important factor to consider, as the elderly tend to suffer from chronic inflammation, as already demonstrated, and drugs can be major modulators of the immune system. Such studies with a long enough follow-up period and a sufficient sample size need to be conducted. There are several confounding factors that future studies must address. For example, many studies did not clearly distinguish between pharmacological antipyresis and external cooling. This is a significant oversight, as the mechanisms by which these two methods reduce fever and the body's response to them are entirely different. Additionally, studies should stratify populations based on the severity and etiology of illness, as different conditions may have different outcomes with antipyretic use. The adverse effects of antipyretics, well-documented in case reports and series, provide important data for immunological research to help determine in which patients such treatment should be avoided. Immunological studies also need to investigate the connection between fever and chronic inflammation as the latest pandemic has given rise to interesting observations, such as the absence of fever and the presence of chronic comorbidities leading to increased mortality from the infection [94]. We need to examine these factors through extensive observational studies and use the results to plan RCTs to evaluate the exact relevance of antipyretic treatment in the elderly. Additionally, physiological and immunological research is necessary to clarify the role of fever in elderly patients with sepsis and to understand what happens during antipyretic treatment.

## Conclusion

In summary, this systematic review and narrative synthesis on the long-term health effects of fever treatment with antipyretic drugs during infections in the elderly found insufficient evidence to draw firm conclusions. Specifically, no evidence was available for the association between fever suppression and chronic inflammatory disease. The evidence regarding fever reduction and mortality was of low quality, and those regarding hemodynamic alterations, length of stay, and adverse events were of very low certainty. Future high-quality studies with sufficient follow-up periods addressing this question are warranted, considering how common the use of antipyretics is in the elderly and how easily this population suffers from the effects of acute inflammatory diseases.

## Data Availability

The datasets generated and/or analyzed during the current study are available as additional material at Mahesh, Seema; Werf, Esther van der; Mallappa, Mahesh; Lai, Nai Ming; Vithoulkas, George (2024). Long-term Health Effects of Antipyretic Drug Use in the Ageing Population: A Systematic Review. Figshare. Dataset. https://doi.org/10.6084/m9.figshare.25650411.v1 and extended data at Mahesh, Seema; Werf, Esther van der; Mallappa, Mahesh; Vithoulkas, George; Lai, Nai Ming (2021). data_SR_antipyretics_elderly. figshare. Dataset. https://doi.org/10.6084/m9.figshare.25650411 Any further data required may be accessed by writing to the corresponding author. **Preprint of this article available at:** https://web.archive.org/web/20211001104029id_/https://assets.researchsquare.com/files/rs-907924/v1/3ac12c2c-de68-4aae-b3f7-ff98f9b47506.pdf?c=1632325421 DOI: https://doi.org/10.21203/rs.3.rs-907924/v1

## Personal thanks

The authors thank Taylors University ageing flagship program for funding this research.
